# Pseudo-Meigs‘syndrome mimicking malignant pleural mesothelioma: a case report and diagnostic pitfall analysis

**DOI:** 10.3389/fmed.2026.1849707

**Published:** 2026-06-17

**Authors:** Hongmei Sheng, Wenkang Zong, Zhenchun Song, Qihong Yu

**Affiliations:** Department of Respiratory and Critical Care Medicine, Tianjin Chest Hospital, Tianjin, China

**Keywords:** case report, diagnostic pitfall, malignant pleural mesothelioma, misdiagnosis, ovarian borderline tumor, pleural effusion, pseudo-Meigs’ syndrome

## Abstract

**Objective:**

To investigate the clinical characteristics, misdiagnosis causes, and preventive strategies of pseudo-Meigs’ syndrome, and to improve clinicians‘ awareness of pelvic tumors presenting with pleural effusion and ascites.

**Methods:**

A 45-year-old woman presented with a 2-month history of dyspnea and a large right-sided pleural effusion with markedly elevated CYFRA 21-1 (>500 ng/mL). Thoracoscopic pleural biopsy showed atypical cells with positive Calretinin, leading to a pathology report of “suspicious for mesothelioma.” The patient was diagnosed with malignant pleural mesothelioma and received two cycles of pemetrexed plus carboplatin chemotherapy. When symptoms did not improve and ascites developed, pelvic CT revealed a 7.0 × 5.5 cm ovarian mass.

**Results:**

Postoperative pathology confirmed bilateral ovarian borderline serous cystadenoma with focal low-grade serous carcinoma—pseudo-Meigs‘ syndrome. After tumor resection, the pleural effusion and ascites resolved completely, and the patient remained disease-free at 4-year follow-up.

**Conclusion:**

Pseudo-Meigs’ syndrome can closely mimic malignant pleural mesothelioma. For any woman with unexplained pleural effusion, pelvic imaging should be performed before invasive thoracic procedures to avoid catastrophic misdiagnosis.

## 1 Introduction

Meigs’ syndrome was classically defined by Meigs and Cass in 1937 as the triad of benign ovarian fibroma (or thecoma), ascites, and hydrothorax, with complete resolution of effusions after tumor resection (1). When the same clinical picture is caused by other ovarian tumors—including other benign tumors, borderline tumors, or even malignancies—the term pseudo-Meigs‘ syndrome has been applied (2).

The clinical importance of recognizing pseudo-Meigs’ syndrome lies in its excellent prognosis after surgical resection, which stands in stark contrast to the poor outcomes of the malignancies it can mimic. Malignant pleural mesothelioma is an aggressive neoplasm with a median survival of less than 12 months, while pseudo-Meigs‘ syndrome is potentially curable with surgery alone (3). The diagnostic challenge is compounded by the fact that tumor markers such as CA-125 and CYFRA 21-1 can be markedly elevated in pseudo-Meigs’ syndrome, further misleading clinicians toward a malignant diagnosis (4, 5). To date, there are only a handful of reported cases of pseudo-Meigs‘ syndrome initially misdiagnosed as malignant pleural mesothelioma, and even fewer with complete histopathological documentation of both the initial misdiagnosis and the final correct diagnosis. Herein, we report a case of pseudo-Meigs’ syndrome secondary to ovarian borderline serous cystadenoma with low-grade serous carcinoma that was initially misdiagnosed as malignant pleural mesothelioma based on our own pleural biopsy pathology, leading to unnecessary chemotherapy. We present this case to highlight the diagnostic pitfalls and to propose a practical algorithm to avoid such catastrophic errors.

## 2 Case presentation

### 2.1 Case selection criteria

This is a retrospective case report. The inclusion criterion was a patient with pathologically confirmed pseudo-Meigs‘ syndrome with complete clinical, imaging, and follow-up data. The exclusion criterion was incomplete clinical data or loss to follow-up. This case met the inclusion criteria.

### 2.2 Patient information and history

A 45-year-old woman presented to our respiratory department on May 20, 2021, with a 2-month history of progressive dyspnea on exertion. Chest CT from an outside hospital revealed a large right-sided pleural effusion, for which a thoracentesis tube had been placed. Tuberculosis had been ruled out. The patient was admitted to our department for further evaluation. Her past medical history was unremarkable. She denied fever, night sweats, hemoptysis, or weight loss. She had no history of smoking or occupational asbestos exposure.

### 2.3 Physical examination and initial investigations

Physical examination: Vital signs were stable. A closed thoracostomy drainage tube was in place on the right side. Breath sounds were markedly diminished on the right but clear on the left. Cardiac, abdominal, and lower extremity examinations were unremarkable.

Laboratory investigations: Complete blood count and liver/kidney function tests were normal. Serum tumor markers: CYFRA 21-1 was 9.11 ng/mL (normal <3.3 ng/mL); serum CA-125 was within the normal range. Pleural fluid analysis: orange-colored, turbid, Rivalta test positive; nucleated cell count 1.8 × 10^9^/L (predominantly mononuclear cells); biochemistry: adenosine deaminase 6.10 U/L, protein 37.20 g/L, lactate dehydrogenase 273.00 U/L; pleural fluid CYFRA 21-1 > 500 ng/mL. Pleural fluid cytology and cell block revealed no definite malignant cells.

### 2.4 Diagnostic workup and initial misdiagnosis

Given the markedly elevated CYFRA 21-1 and the suspicion of malignant pleural effusion, a medical thoracoscopy was performed. Intraoperatively, diffuse miliary nodules and extensive adhesions were observed on both the parietal and visceral pleura. Pleural biopsy was taken from the parietal pleura. Histopathology (Figure 1) revealed: adipose tissue and proliferative fibrous connective tissue, abundant inflammatory granulation tissue with necrosis, and scattered cords of atypical cells. Immunohistochemistry showed: Calretinin (+), CK(P) (+), WT-1 (+), Vimentin (+); Ki-67 was >10%. Special staining (Masson‘s trichrome) confirmed the findings. The pathology report concluded: “Suspicious for mesothelioma.” Based on this report, the patient was diagnosed with malignant pleural mesothelioma and received two cycles of combination chemotherapy with pemetrexed (0.8 g) plus carboplatin (0.4 g) on June 1 and June 24, 2021.

**FIGURE 1:**
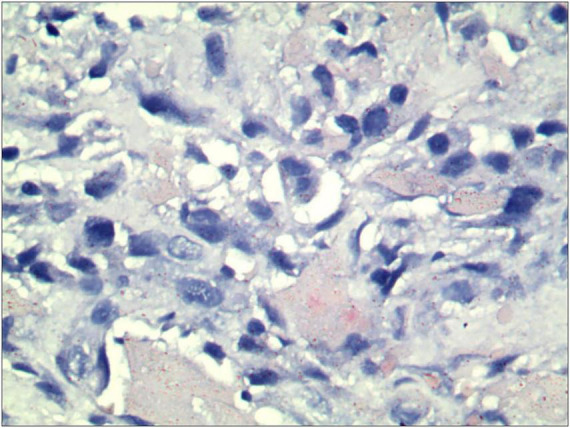
Pleural biopsy pathology (initial misdiagnosis). Hematoxylin and eosin staining (original magnification ×400) showing scattered cords of atypical cells within inflammatory granulation tissue and fibrous connective tissue. The pathology report concluded “suspicious for mesothelioma” based on atypical morphology and positive Calretinin immunohistochemistry.

### 2.5 Clinical deterioration and diagnostic reassessment

After two cycles of chemotherapy, the patient reported no improvement in her dyspnea. Furthermore, she developed new symptoms including abdominal distension, anorexia, and fatigue. Abdominal ultrasound revealed moderate ascites and a hypoechoic solid mass in the lower abdomen. Contrast-enhanced pelvic CT was then performed, and the report revealed: irregular soft tissue masses and nodular opacities with heterogeneous enhancement in the uterus and bilateral adnexal regions, highly suspicious for malignant neoplasm. Serum CA-125 was repeated and found to be markedly elevated at 696.80 U/mL.

### 2.6 Final diagnosis and treatment outcome

The patient was referred to the gynecology department and underwent exploratory laparotomy. Postoperative histopathology (Figure 2) confirmed: (bilateral) ovarian borderline serous cystadenoma with focal areas transforming into low-grade serous carcinoma, with endometriosis and psammoma bodies. Surgical margins were negative. Following tumor resection, the patient‘s pleural effusion and ascites resolved completely without any further intervention. A follow-up chest CT performed on December 21, 2021, confirmed complete resolution of the right-sided pleural effusion. The entire diagnostic and therapeutic journey is summarized in Figure 3. At 4-year follow-up (December 2021 to November 2025), the patient remains asymptomatic with no evidence of recurrence. A timeline of key clinical events is provided in Figure 4.

**FIGURE 2:**
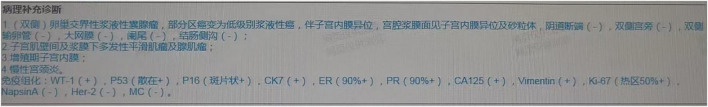
Ovarian pathology (final diagnosis). Hematoxylin and eosin staining showing papillary architecture characteristic of borderline serous cystadenoma. Higher magnification showing mild nuclear atypia without stromal invasion, consistent with borderline tumor with low-grade carcinoma.

**FIGURE 3:**
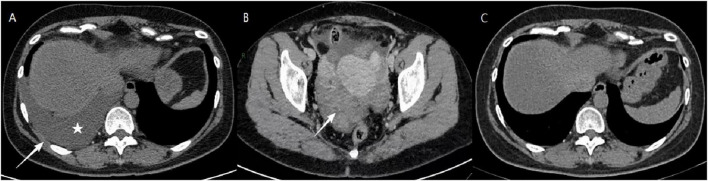
Imaging comparison before and after treatment. **(A)** Chest CT at admission showing a large right-sided pleural effusion (⋆) with pleural nodular thickening (→). **(B)** Contrast-enhanced pelvic CT showing irregular soft tissue masses and nodular opacities with heterogeneous enhancement in the uterus and bilateral adnexal regions (→), highly suspicious for malignant neoplasm. **(C)** Chest CT 4 months after ovarian tumor resection showing complete resolution of the pleural effusion.

**FIGURE 4:**
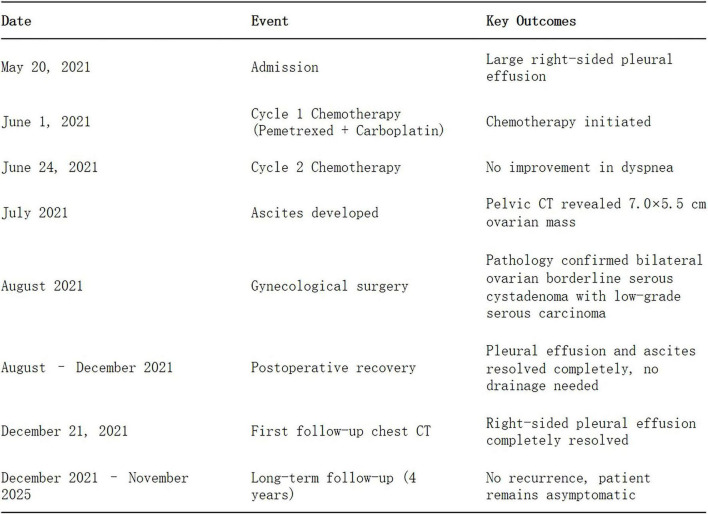
Timeline of key clinical events from admission to 4-year follow-up, including diagnostic steps, therapeutic interventions, and outcomes.

## 3 Discussion

This case illustrates a catastrophic diagnostic pitfall: a curable condition (pseudo-Meigs’ syndrome) was initially misdiagnosed as an incurable malignancy (malignant pleural mesothelioma), leading to unnecessary chemotherapy. We analyze the reasons for this misdiagnosis and propose practical strategies for prevention.

### 3.1 Pseudo-Meigs‘ syndrome: definition and diagnostic criteria

Pseudo-Meigs’ syndrome is defined by the following criteria: (1) presence of an ovarian tumor (which may be benign, borderline, or malignant, excluding fibroma/thecoma), (2) ascites and/or pleural effusion, and (3) resolution of effusions after tumor resection (2, 6). Our patient met all three criteria: she had an ovarian borderline tumor with low-grade carcinoma, presented with pleural effusion and ascites, and experienced complete resolution after surgery. The pathogenesis of effusions in Meigs and pseudo-Meigs syndromes is not fully understood but may involve mechanical irritation, lymphatic obstruction, or secretion of inflammatory cytokines such as vascular endothelial growth factor (7).

### 3.2 Reasons for misdiagnosis

#### 3.2.1 Cognitive bias: anchoring and confirmation bias

The patient presented with respiratory symptoms, and all initial investigations (imaging, pleural fluid analysis) pointed to a thoracic etiology. This created a strong anchoring bias—once “thoracic malignancy” was anchored, subsequent clinical reasoning tended to seek confirming evidence and filter out disconfirming information (8). The failure to follow the basic principle—“In any woman with unexplained pleural effusion, pelvic imaging should be considered mandatory”—was the most fundamental management error in this case (9).

#### 3.2.2 Histopathological pitfalls

The pleural biopsy report stated “suspicious for mesothelioma,” which inherently conveys uncertainty. However, this uncertainty was overlooked. Reactive mesothelial hyperplasia can be exceedingly difficult to distinguish from malignant mesothelioma, with reported misdiagnosis rates of 10%–15% even among experienced pathologists (10). In retrospect, several features should have raised suspicion: the presence of abundant inflammatory granulation tissue and necrosis is more typical of reactive processes than mesothelioma. A second pathology opinion or multidisciplinary discussion might have prevented the misdiagnosis.

#### 3.2.3 Misleading tumor markers

The markedly elevated CYFRA 21-1 (>500 ng/mL) in pleural fluid was a powerful driver of the misdiagnosis. However, CYFRA 21-1 is not 100% specific for malignancy. Elevated levels have been documented in Meigs and pseudo-Meigs syndromes, likely due to mesothelial cell stimulation and proliferation (4, 5, 11). A similar diagnostic challenge was reported by Iavarone et al., who described a case of Meigs syndrome with markedly elevated CA-125 mimicking ovarian cancer (12). Their case reinforces the principle that elevated tumor markers (both CA-125 and CYFRA 21-1) are not specific for malignancy in the setting of Meigs or pseudo-Meigs syndromes. This case reinforces the principle that no single biomarker should be used in isolation to diagnose malignancy.

### 3.3 Learning points and clinical recommendations

Based on this case, we propose the following practical recommendations:

#### 3.3.1 Mandatory pelvic screening in women with unexplained pleural effusion

Pelvic ultrasound or CT should be performed before invasive thoracic procedures (thoracoscopy, pleural biopsy) in any woman with unexplained pleural effusion. This simple, low-cost, non-invasive test could have identified the ovarian tumor early and prevented the unnecessary thoracoscopy and chemotherapy.

#### 3.3.2 Recognize the histopathological mimicry

Reactive mesothelial hyperplasia can closely mimic malignant mesothelioma. When a pleural biopsy shows “atypical mesothelial proliferation” with inflammatory features, the differential diagnosis should always include Meigs/pseudo-Meigs syndrome in a woman with an ovarian mass.

#### 3.3.3 Biomarkers are not diagnostic

Markedly elevated CYFRA 21-1 in pleural fluid is suggestive but not diagnostic of malignancy. This finding should prompt further investigation, but not definitive treatment, without confirmatory evidence.

### 3.4 Comparison with previously reported cases

A literature search reveals only a handful of cases of pseudo-Meigs‘ syndrome misdiagnosed as malignant pleural mesothelioma. To our knowledge, this is one of the few reports that provides complete histopathological documentation of both the initial misdiagnosis (pleural biopsy) and the final correct diagnosis (ovarian pathology), making it a uniquely valuable teaching case. In 2024, Liu et al. conducted a systematic review of misdiagnosed pseudo-Meigs’ syndrome cases and found that over 60% of cases were initially misdiagnosed as advanced malignancies, with ovarian cancer and mesothelioma being the most common misdiagnoses (13). In 2025, Kim et al. reported a case of pseudo-Meigs‘ syndrome mimicking pleural mesothelioma, with a clinical course highly similar to our case, further confirming the prevalence of this diagnostic pitfall (14).

### 3.5 Strengths of this study

This case report has several strengths. First, it provides complete histopathological documentation of both the initial misdiagnosis (pleural biopsy) and the final correct diagnosis (ovarian pathology), which is rarely available in previously reported cases. Second, the 4-year follow-up confirms the durability of the curative outcome after tumor resection. Third, the three learning points derived from this case offer practical, actionable guidance for clinicians to avoid similar diagnostic pitfalls.

## 4 Conclusion

Pseudo-Meigs‘ syndrome is a great mimicker of malignant pleural mesothelioma. We report a case where this diagnostic pitfall led to unnecessary chemotherapy. The key lessons are: (1) pelvic imaging should be mandatory in any woman with unexplained pleural effusion, (2) the histopathological distinction between reactive mesothelial hyperplasia and mesothelioma can be challenging and requires caution, and (3) elevated CYFRA 21-1 is not specific for malignancy. Awareness of this syndrome and adherence to a systematic diagnostic approach can prevent catastrophic misdiagnosis and ensure potentially curative surgery for affected patients.

## Data Availability

The data analyzed in this study is subject to the following licenses/restrictions: The datasets presented in this article are not publicly available because they consist of confidential patient medical records. Access to these data is restricted to protect patient privacy. Requests to access the datasets should be directed to the corresponding author and will require approval from the institutional ethics committee and a data sharing agreement. Requests to access these datasets should be directed to QY, yuqihong1109@126.com.
